# Relationship Between Kindergarten Organizational Climate and Teacher Burnout: Work–Family Conflict as a Mediator

**DOI:** 10.3389/fpsyt.2020.00408

**Published:** 2020-05-15

**Authors:** Dongying Ji, Yaping Yue

**Affiliations:** ^1^Faculty of Education, Beijing Normal University, Beijing, China; ^2^School of Education Science, Henan University, Kaifeng, China

**Keywords:** kindergarten organizational climate, kindergarten teacher, burnout, work–family conflict, mediating effect

## Abstract

Burnout in kindergarten teachers is influenced by individual factors, social factors, and organizational factors. Kindergarten organizational climate as an external work resource may cause teacher burnout when it cannot meet their work demands. To explore the mechanisms that underlie the effects of kindergarten organizational climate on teacher burnout, we investigated the mediating effect of work-family conflict (i.e., work interfering with family and family interfering with work) on the relationship between kindergarten organizational climate and teacher burnout. The study sample included 436 kindergarten teachers in Henan, China. The Chinese versions of the Kindergarten Organizational Climate Scale, Kindergarten Teachers Work-Family Conflict Scale, and Kindergarten Teachers Burnout Scale were applied. The results showed that kindergarten organizational climate was positively correlated with work–family conflict and teacher burnout. Work–family conflict was positively correlated with teacher burnout. Work–family conflict partially mediated the effects of kindergarten organizational climate on teacher burnout. The mediating effect of family interfering with work was significantly stronger than the mediating effect of work interfering with family. The results are discussed with respect to the general literature on the correlation between organizational climate, WFC, and burnout.

## Introduction

Burnout represents a state of physical, mental, and emotional exhaustion that is caused by work stress (1, 2). It is generally thought to consist of three dimensions: emotional exhaustion, dehumanization, and a reduction of personal accomplishment (3). Teacher burnout refers to a negative reaction when a teacher is unable to cope with work-related stress. It is a state of emotional, attitudinal, and behavioral decline that is caused by a teacher's long-term experience with stress (4). Kindergarten teachers not only care for and teach children between the ages three and six, but also provide them with warm, nurturing and learning experience, and lay the foundation for their future social, emotional and academic success (5). Therefore, kindergarten teachers need to put in more effort to care for children, which is easy to lead to physical and mental fatigue (6). Kindergarten teachers also experience tremendous pressure from children's parents (7), so they are more likely to suffer from burnout.

Organizational climate is often considered as relatively temporary, subject to direct control, and largely limited to those aspects of the social environment that are consciously perceived by organizational members (8). Its core focuses on the description of how organizational influences impact organizational members (9). Cropanzano et al. (10) reported perceived organizational support to be negatively related to burnout. Kanste, Kynga and Nikkila (11) found that support and respect from the supervisor protect organizational members from job burnout. Lee (12) found significant direct effects of a positive organizational climate predicting lower levels of burnout (12). Boamah et al. (13) also found that the adequate superior support was an effective resource for organizational members to relieve work pressure and reduce burnout. Kindergarten, as an organization, also relies on the teachers as the key employees, so the teacher's perception of the kindergarten's work environment is the core of kindergarten organizational climate (9). The organizational climate is a bridge between the kindergarten organizational system and teacher behavior. Based on the Job Demand–Resources Model, the kindergarten organizational climate belongs to an external work resource that can cause teacher burnout when it is unbalanced with work demand (14). A study of kindergarten teacher burnout in Beijing showed that organizational factors have a stronger impact on kindergarten teacher burnout compared with social factors, professional factors, and personal factors (15). Discussing kindergarten organizational climate as an antecedent of kindergarten teacher burnout at the organizational level is meaningful.

Work–family conflict (WFC) is experienced when demands of one role in one domain interfere with participation in or the performance of the other role (16). Compared with men, women experience more family interfering with work (FIW) and strain-based work interfering with family [WIF; (17)]. Female-dominated kindergarten teachers experience even more WFC. Even they have paid work, women have not relinquished the demands of their families (18). Work–family conflict is closely correlated with organizational factors, and increasingly more people realize that organizational climate plays an important role in the balance between work and family (19–21). Organizational support is considered one of the most effective factors in reducing WFC (22). WFC (includes WIF, FIW) is an important antecedent of burnout and has a significant impact on burnout (23, 24). Demerouti et al. stipulated a kind of “spiral relationship” between WFC and burnout. They found that WFC caused burnout among employees, and burnout resulted in a decline of employees' work performance. Thus, burnout aggravated WFC, and WFC generated higher burnout (14). Work–family conflict is clearly not only impacted by organizational climate—it also significantly impacts burnout. Additionally, some studies have considered that WFC might also act as a mediator of the relationship between work demand and turnover intention (25), job satisfaction (26), and happiness (27).

Frone (28) indicated that WFC was a bidirectional concept that consists of WIF and FIW. The two types of WFC have been examined in many studies. The degree of WIF that was perceived by adults was stronger than their perceived FIW (23, 24, 29–31). Both concepts, WIF and FIW, exert different effects on outcome variables. Some studies found that the predictive effect of WIF on turnover intention was stronger than FIW (32). The correlation between FIW and self-efficacy was significantly stronger compared with WIF (33). Work interfering with family had a significant impact on teachers' mental health, whereas FIW had no significant impact on teachers' mental health (34). The mediating effects of WIF and FIW have also been examined. Ford et al. (35) performed a meta-analysis of Frone's Work-Family Conflict Model and found that WIF partially mediated the relationship between antecedents of work factors and family satisfaction, whereas the mediating effect of FIW was not proven. Furthermore, Nie and Xie (36) tested a multiple mediator model of bidirectional WFC in a sample of 413 employees from 28 provinces in China. They found that WIF mediated the relationship between family-supportive supervisor behaviors and job satisfaction, whereas FIW did not.

Little research has explored work-family conflict of kindergarten teachers and its mediating effect between organizational climate and teacher burnout. This study hopes to expand the research on the work–family conflict, focuses on kindergarten teachers in particular to explore mediating role of WFC. The main purpose of this study is to examine the relationship between kindergarten organizational climate and teacher burnout, investigate the mediating role of WFC, WIF, and FIW in the relationship between kindergarten organizational climate and teacher burnout, further compare the mediating effects of WIF and FIW. Specifically, the study hypotheses are as follows:

Hypothesis 1: There are significant correlations between kindergarten organizational, WFC, and teacher burnout.
Hypothesis 2: Work–family conflict mediates the relations between kindergarten organizational climate and teacher burnout.

## Materials and Methods

### Participants and Procedure

The participants were teachers from kindergartens in Henan province, China. About 30 kindergartens participated in this study, each kindergarten has 9–15 teaching classes. For example, some kindergartens have nine classes, some kindergartens have 12 classes, and others have 15 classes. Classes are guided by two professional teachers and one nurse. Thus, each of the participating kindergartens employs 18 to 30 professional teachers. This study focuses on the professional teachers, we did not approach nurses for study participation. The heads of these kindergartens were approached, and consent was obtained before conducting the survey. After obtaining informed consent to conduct this survey, the kindergarten managers distributed the questionnaires to the teachers in 2017. The questionnaires were then collected within 2 weeks. A total of 485 questionnaires were administered to the kindergarten teachers, of which 450 were returned, indicating a response rate of 92.8%. Four hundred thirty-six of these 450 questionnaires were valid, indicating an effective rate of 89.9%. These 436 responses formed the final sample. The procedures followed were in accordance with the ethical standards of the Committee on Medical and Scientific Research of Medical College of Henan University. **Table 1** presents the demographic characteristics of the sample.

**Table 1:** Demographic information of kindergarten teacher sample (*n* = 436).

Characteristics	*n* (%)
**Kindergarten type**	
Public	229 (52.5)
Private	207 (47.5)
**Gender**	
Male	7 (1.6)
Female	429 (98.2)
**Age**	
Under 20	45 (10.3)
20–29	272 (62.2)
30–39	97 (22.2)
40 and above	22 (5.1)
**Education level**	
≤High school	45 (10.3)
Vocational school	243 (55.7)
≥University degree	148 (33.9)
**Teaching experience**	
1–5 years	274 (62.8)
6–10 years	92 (21.1)
11–15 years	41 (9.4)
16–20 years	17 (3.9)
>20 years	12 (2.8)

### Organizational Climate Scale for Kindergartens

The Organizational Climate Scale for Kindergartens (37–39) was used to assess the teacher-perceived organizational climate of their kindergartens. The scale was adapted from the Organizational Climate Scale that was originally validated by Denison and Mishra (40). The scale was designed to assess four components of organizational climate that were perceived by kindergarten teachers: involvement (e.g., most teachers can participate in the decision-making and planning of the development of the kindergarten), consistency (e.g., most teachers' approach to doing work in the kindergarten is very consistent and predictable), adaptability (e.g., the kindergarten's decision-making and development are flexible and could be adjusted according to the situation), mission (e.g., kindergarten has long-term planning and development direction). The scale consisted of 60 items. All of the items were scored on a 5-point Likert-type scale, ranging from 1 (strongly disagree) to 5 (strongly agree). Items 15, 24, 29, 34, 39, 50, 60 will be scored in reverse. The higher the score of four dimensions, the better the organizational climate perceived by kindergarten teachers. In the present study, Cronbach's α was 0.90. Internal consistency was 0.81 for support climate, 0.86 for intimacy climate, 0.91 for adaptability climate, and 0.84 for development climate.

### Maslach Burnout Inventory

The Maslach Burnout Inventory (MBI) was developed by Maslach & Jackson and has three versions: MBI-Human Service Survey (MBI-SS), MBI-Educators Survey (MBI-ES), and MBI-General Survey [MBI-GS; (41)]. Among them, the MBI-ES has been reported to have good cross-cultural reliability and validity and is the most widely used measurement instrument for teacher burnout (42). The determination of kindergarten teacher burnout was adapted from the MBI-ES to fit the kindergarten teacher context. The MBI, consisting of 22 items, was designed to assess three components of burnout among kindergarten teachers: emotional exhaustion, depersonalization, and personal accomplishment. All of the items were scored on a 7-point Likert-type scale, ranging from 1 (never) to 7 (always). Items 4, 7, 8, 9, 12, 14, 17, 18 and 19 will be scored in reverse. The higher the score of three dimensions, the higher the degree of teacher burnout. In the present study, Cronbach's α was 0.75. Internal consistency was 0.87 for emotional exhaustion, 0.86 for depersonalization, and 0.68 for personal accomplishment.

### Kindergarten Teacher Work–Family Conflict Scale

The Kindergarten Teacher Work–Family Conflict Scale was used to assess teacher-perceived conflict between kindergartens and their families. In the present study, the scale was adapted from the Teacher Work–Family Conflict Scale that was validated by Wu et al. (43), which was developed on the basis of the two directions (WIF and FIW) and three forms of work–family conflict (behavior-based conflict, time-based conflict, and stress-based conflict) that were proposed by Greenhaus et al. (16). The scale consisted of 22 items. All of the items were scored on a 5-point Likert-type scale, ranging from 1 (strongly disagree) to 5 (strongly disagree). Higher values indicate higher levels of work–family conflict. In the present study, Cronbach's α was 0.97. Internal consistency was 0.88 for WIF and 0.84 for FIW.

### Data Analysis

The data were analyzed using PASW 18.0 software. Bivariate correlations were calculated to measure correlations between kindergarten organizational climate, WFC (WIF, FIW), and teacher burnout. The role of WFC, WIF, and FIW in mediating the association between kindergarten organizational climate and teacher burnout was tested by Bootstrap. Bootstrap and mediating model 4 were used in the present study. The “resampling size” was set at 1,000, which implied that the unstandardized indirect effect of the mediation path was computed for every 1,000 bootstrapped samples. The confidence interval (CI) was set at 95%, which implied that the indirect effects at the 2.5th and 97.5th percentiles were determined by computing a 95% CI. A statistically significant mediating effect was confirmed if the CI of the indirect path did not include 0 (44).

## Results

### Correlation Analysis

The means, standard deviations, and intercorrelations among variables that were evaluated in the present study are presented in **Table 2**. The results showed that kindergarten organizational climate was negatively correlated with teacher burnout (*r* = −0.55, *p <*0.01), WFC (*r* = −0.25, *p <*0.01), WIF (*r* = −0.12, *p <*0.05), and FIW (*r* = −0.32, *p <*0.01). Work-family conflict, WIF, and FIW were positively correlated with teacher burnout (*r_WFC_* = 0.48, *r_WIF_* = 0.33, *r_FIW_* = 0.50, all *p <*0.01).

**Table 2:** Means, standard deviations, and intercorrelations of the observed variables.

Variable	Mean	SD	1	2	3	4	5
1. KOC	3.73	0.48	–				
2. TB	2.69	0.76	−0.55***	–			
3.WFC	2.60	0.66	−0.29***	0.49***	–		
4. WIF	2.92	0.82	−0.15**	0.34***	0.87***	–	
5. FIW	2.28	0.72	−0.36***	0.51***	0.84***	0.46***	–

Pearson correlations for kindergarten teachers (n = 436) are presented above the diagonal. **p < 0.01, ***p < 0.001. KOC, kindergarten organizational climate; TB, teacher burnout; WFC, work–family conflict; WIF, work interfering with family; FIW, family interfering with work.

### Mediating Effect Analysis

Significant correlations were found between kindergarten organizational climate, WFC, and teacher burnout. We have hypothesized that work–family conflict plays a mediating role between kindergarten organizational climate and teacher burnout. Therefore, we further tested the mediating role of WFC (see **Supplementary Datasheet 1**).

In this mediating model, kindergarten organizational climate and WFC explained 43.69% of the variance of teacher burnout. Kindergarten organizational climate explained 1.51% of the variance of WIF. Kindergarten organizational climate explained 9.96% of the variance of FIW.

### Mediating Effect of WFC

**Table 3** shows that the direct path from kindergarten organizational climate to teacher burnout was significant (95% CI = [−0.92, −0.69], *t* = −13.89, *p <* 0.001) in a model that did not include WFC. The direct path between kindergarten organizational climate and teacher burnout remained significant (95% CI = [−0.76, −0.54], *t* = −11.89, *p <*0.001) when WFC was included in the model, indicating that WFC partially mediated the association between kindergarten organizational climate and teacher burnout.

**Table 3:** Mediating effect of WFC.

	Effect	SE	*t*	*p*	LLCI	ULCI
KOC → TB (c)	−0.80	0.06	−13.89	0.000	−0.92	−0.69
KOC → WFC (a)	−0.39	0.06	−6.28	0.000	−0.51	−0.27
WFC → TB (b)	0.39	0.04	9.55	0.000	0.31	0.47
KOC →TB (c')	−0.65	0.06	−11.89	0.000	−0.76	−0.54
KOC → WFC → TB	−0.15	0.03			−0.22	−0.10

LLCI, the lowest value of the confidence interval; ULCI, The highest value of the confidence interval; c, the total effect of kindergarten organizational climate on teacher burnout; a, the effect of kindergarten organizational climate on work–family conflict; b, the effect of work–family conflict on teacher burnout; c', the direct effect of kindergarten organizational climate on teacher burnout after the introduction of work–family conflict; KOC, kindergarten organizational climate; TB, teacher burnout; WFC, work–family conflict.

### Mediating Effect of WIF

**Table 4** shows that the direct path from kindergarten organizational climate to teacher burnout was significant (95% CI = [−0.92, −0.69], *t* = −13.89, *p <*0.001) in a model that did not include WIF. The direct path between kindergarten organizational climate and teacher burnout remained significant (95% CI = [−0.86, −0.64], *t* = −13.43, *p <*0.001) when WIF was included in the model, indicating that WIF partially mediated the association between kindergarten organizational climate and teacher burnout. The bootstrapped standardized indirect effect on the path from kindergarten organizational climate to teacher burnout was significantly different from 0. The mediating effect of WIF was −0.06 (a_1__*_ b_1_).

**Table 4:** Mediating effect of WIF.

	Effect	SE	*t*	*p*	LLCI	ULCI
KOC→ TB (c)	−0.80	0.06	−13.89	0.000	−0.92	−0.69
KOC → WIF (a_1_)	−0.24	0.08	−3.14	0.002	−0.40	−0.09
WIF → TB (b_1_)	0.24	0.04	6.94	0.000	0.17	0.30
KOC → TB (c'_1_)	−0.75	0.06	−13.43	0.000	−0.86	−0.64
KOC → WIF → TB	−0.06	0.02			−0.10	−0.02

LLCI, the lowest value of the confidence interval; ULCI, The highest value of the confidence interval; c, the total effect of kindergarten organizational climate on teacher burnout; a_1_, the effect of kindergarten organizational climate on work interfering with family; b_1_, the effect of work interfering with family on teacher burnout; c'_1_, the direct effect of kindergarten organizational climate on teacher burnout after the introduction of work interfering with family; KOC, kindergarten organizational climate; TB, teacher burnout; WIF, work interfering with family.

### Mediating Effect of FIW

**Table 5** shows that the direct path from kindergarten organizational climate to teacher burnout was significant (95% CI = [−0.92, −0.69], *t* = −13.89, *p <* 0.001) in a model that did not include FIW. The direct path between kindergarten organizational climate and teacher burnout remained significant (95% CI = [−0.73, −0.51], *t* = −10.93, *p <* 0.001) when FIW was included in the model, indicating that FIW partially mediated the association between kindergarten organizational climate and teacher burnout. The bootstrapped standardized indirect effect on the path from kindergarten organizational climate to teacher burnout was significantly different from 0. The mediating effect of FIW was −0.18 (a_2__*_ b_2_).

**Table 5:** Mediating effect of FIW.

	Effect	SE	*t*	*p*	LLCI	ULCI
KOC → TB (c)	−0.80	0.06	−13.89	0.000	−0.92	−0.69
KOC → FIW (a_2_)	−0.53	0.07	−7.92	0.000	−0.66	−0.40
FIW → TB (b_2_)	0.35	0.04	9.06	0.000	0.27	0.42
KOC → TB (c'_2_)	−0.62	0.06	−10.93	0.000	−0.73	−0.51
KOC → FIW → TB	−0.18	0.03			−0.25	−0.13

LLCI, the lowest value of the confidence interval; ULCI, The highest value of the confidence interval; c, the total effect of kindergarten organizational climate on teacher burnout; a_2_, the effect of kindergarten organizational climate on family interfering with work; b_2_, the effect of family interfering with work on teacher burnout; c'_2_, the direct effect of kindergarten organizational climate on teacher burnout after the introduction of family interfering with work; KOC, kindergarten organizational climate; TB, teacher burnout; FIW, family interfering with work.

### Comparison of the Mediating Effects of WIF and FIW

**Figure 1:**
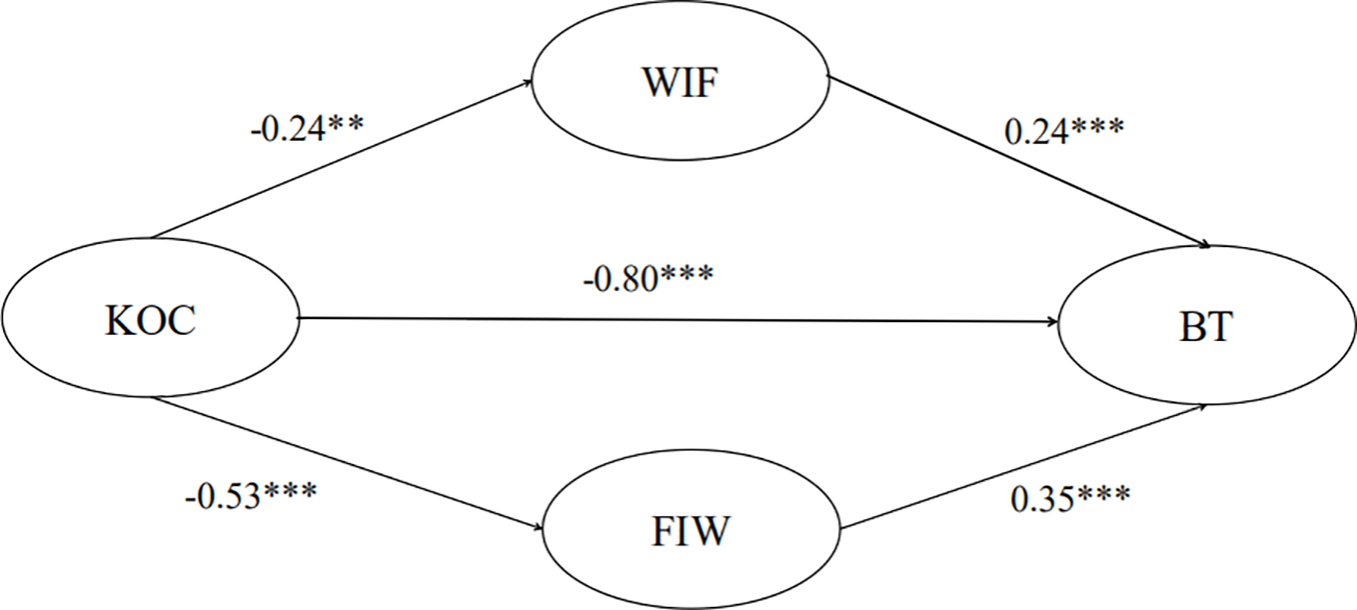
Model of the relationship among kindergarten organizational climate, WIF, FIW, and teacher burnout. KOC, kindergarten organizational climate; TB, teacher burnout; WIF, work interfering with family; FIW, family interfering with work. **p < 0.01, ***p < 0.001.

The model of the relationship among kindergarten organizational climate, WIF, FIW, and teacher burnout is presented in **Figure 1**. The mediating effect of WIF was −0.06 (−0.24 ∙ 0.24), and the mediating effect of FIW was −0.18 (−0.53 ∙ 0.35). This implied that the mediating effect of FIW on the relationship between kindergarten organizational climate and teacher burnout was stronger than the mediating effect of WIF (the sign only represents positive and negative effects) on the relationship between kindergarten organizational climate and teacher burnout. Compared with WIF, kindergarten organizational climate had more of an influence on teacher burnout through FIW.

## Discussion

The present study investigated the correlation between kindergarten organizational climate, WFC, and teacher burnout. Kindergarten organizational climate was negatively correlated with teacher burnout. This finding is consistent with previous studies (45, 46), which reported that school organizational climate had a significant impact on burnout. A higher degree of non-supportive school organizational climate was associated with a stronger teachers' sense of stress and burnout. The present study also found that kindergarten organizational climate was significantly negatively correlated with the teachers' WFC, which was consistent with the conclusion of a previous study (47) that included a sample of clinical nurses. Both kindergarten teachers and clinical nurses engage in a nurturing profession and inevitably suffer conflicts between work and family. A supportive organizational climate can serve as a potential force to alleviate conflict when it arises. The present study found that WFC was positively correlated with teacher burnout, which was consistent with previous studies (48–50). Bagherzadeh et al. (24) reported that there was a significantly negative association between WIF and overall burnout, FIW was significantly associated with depersonalization.

The present study found WFC mediated the relationship between kindergarten organizational climate and teacher burnout. The previous studies have focused more on the relationship between organizational climate and WFC or WFC and burnout. The relationship between the three constructs has not been reported in previous studies. According to Work-Family Boundary Theory, boundaries between work and family have certain permeability and flexibility. Permeability and flexibility determine the boundary strength between work and family (51). The greater the flexibility of the borders between work and home, the lower the level of work–family conflict (52). However, the permeability and flexibility of a weak boundary is high. If the boundary of kindergarten in the work domain is weak, then it is easy to be permeated by the role of the family domain and easier to adjust flexibly to meet the demands of family (51), thus alleviating possible conflict between family and work. Organizational support contributes to better flexibility of the work boundary, and instrumental support resources from managers can provide employees with the ability to engage in a flexible work boundary (53). Mansour (54) pointed out that supervisor support can permit employees to have more resources in work or family. When employees are faced with high family demands, in order to reduce the loss of family resources or to gain more resources, they will try to obtain support from their superiors. For instance, a teacher can take children to school and be a little late for work because of the supervisor's support for his family. WFC significantly positive effects burnout (50, 55), results in the emergence of emotional exhaustion, decreased response to others, and lack of performance (56). Thus, WFC is not only impacted by the organizational climate, but also has a significantly positive effect on burnout.

The present results indicated that the mediating effect of FIW was stronger than the mediating effect of WIF. This implies that WIF and FIW both have a significant impact on burnout, but the effect of FIW on burnout is stronger compared with WIF, consistent with domain specificity (cross-domain) perspective, which suggests that WIF predominantly influences the family, while FIW affects the work. These findings are consistent with previous studies (57, 58). The previous study pointed that organizational support contributes to better flexibility of the work boundary (53). Support from supervisors can act as the “passageways”, which permit employees to have more resources in the family domain (54). When employees feel that the organization cares about them and their family, they invest more resources at home which can reduce stress due to FIW. Barnett et al. (59) found that FIW completely mediated the effect of work demands on distress. Especially, in a collectivist society like China, work is viewed primarily as contributing to family welfare (60). Hard work is thus the main way to promote happiness of the family and fulfill family responsibilities and may bring glory and wealth to the family (61). When teachers are engaged in more family obligations that may interfere with their development demands in work, they are more likely to feel stressed as they struggle to meet job-related demands, which may in turn result in burnout (58). Meanwhile, China's collectivism advocates that people “sacrifice individual interests for public collective benefits” (62). The whole society, including organizations, usually praises the dedication of people who sacrifice their families for work (63). Thus, work that interferes with family is considered normal. Therefore, the influence of WIF on teacher burnout is not as strong as the influence of FIW on teacher burnout.

### Limitations

This study has several limitations that need highlighting. First, the sample of 436 teachers was limited to urban kindergartens in three areas of Henan Province, which reduces the generalizability of the results. Future studies should include more geographic areas and possibly expand to the whole country. Rural kindergartens should also be involved in such studies to examine differences between urban and rural areas. Second, similar to other empirical studies, the present study used cross-sectional data at a certain point in time, which did not clarify the causality of variables. A cross-lagged panel correlation paradigm and longitudinal study should be used to further validate the effects of kindergarten organizational climate and work–family conflict on teacher burnout. Third, the present study only investigated organizational factors rather than family factors as antecedents of work–family conflict. It needs further investigation that which family factors are chief contributors in the conflict. Fourth, we did not delve deeply into the mechanism by which kindergarten organizational climate impacts teacher burnout. We only introduced WFC, but other mediating or moderating variables are likely involved in the effect of organizational climate on teacher burnout.

## Conclusion

To conclude, this study fills the gap, which focused mostly on kindergarten teacher work-family conflict literature, and enriches our knowledge of the relationships between organizational climate, WFC and burnout to the context of Henan, the most populous province in China. We use Work-Family Boundary Theory and Chinese collectivism social culture to explain the mediating effect of WFC and comparison on the mediating effects of WIF and FIW. Effect difference of WIF and FIW on other outcome variables deserves follow-up research and more in-depth discussion.

## Data Availability Statement

The datasets generated for this study are available on request to the corresponding author.

## Ethics Statement

This study was carried out in accordance with the recommendations of the Ethics Committee of Medical College of Henan University on Medical and Scientific Research with written informed consent from all subjects. All subjects gave written informed consent in accordance with the Declaration of Helsinki. The protocol was approved by the Ethics Committee of Medical College of Henan University on Medical and Scientific Research.

## Author Contributions

DJ and YY were responsible for the design of the study. DJ was responsible for the data analysis and writing of the manuscript. YY was responsible for revising of the manuscript.

## Funding

The study was supported by the National Education Science Planning Project of China (BHA170142).

## Conflict of Interest

The authors declare that the research was conducted in the absence of any commercial or financial relationships that could be construed as a potential conflict of interest.

## Supplementary Material

Datasheet 1 The original data.
