# Supplementary material for: Relationship Between Kindergarten Organizational Climate and Teacher Burnout: Work–Family Conflict as a Mediator
Source: Front Psychiatry. 2020 May 15;11:408. doi: 10.3389/fpsyt.2020.00408 (PMC7242754; doi:10.3389/fpsyt.2020.00408)
Supplement: Datasheet 1 — The original data. [file DataSheet_1.pdf]

Correlation

Correlations

		KOC	TB	WFC	WIF	FIW
KOC	Pearson Correlation	1	-.555**	-.289**	-.149**	-.355**
	Sig. (2-tailed)		.000	.000	.002	.000
	N	436	436	436	436	436
TB	Pearson Correlation	-.555**	1	.492**	.343**	.507**
	Sig. (2-tailed)	.000		.000	.000	.000
	N	436	436	436	436	436
WFC	Pearson Correlation	-.289**	.492**	1	.870**	.839**
	Sig. (2-tailed)	.000	.000		.000	.000
	N	436	436	436	436	436
WIF	Pearson Correlation	-.149**	.343**	.870**	1	.462**
	Sig. (2-tailed)	.002	.000	.000		.000
	N	436	436	436	436	436
FIW	Pearson Correlation	-.355**	.507**	.839**	.462**	1
	Sig. (2-tailed)	.000	.000	.000	.000	
	N	436	436	436	436	436

**. Correlation is significant at the 0.01 level (2-tailed).

Mediating effect of WFC

Run MATRIX procedure:

***************** PROCESS Procedure for SPSS Release 2.13 ***************

Written by Andrew F. Hayes, Ph.D. www.afhayes.com
Documentation available in Hayes (2013). www.guilford.com/p/hayes3

****
Model = 4
Y = TB
X = KOC
M = WFC

Sample size
436

****
Outcome: WFC

Model Summary

R	R-sq	MSE	F	df1	df2	p
.2886	.0833	.3929	39.4446	1.0000	434.0000	.0000

Model

	coeff	se	t	p	LLCI	ULCI
constant	4.0514	.2333	17.3668	.0000	3.5929	4.5099
KOC	-.3855	.0614	-6.2805	.0000	-.5061	-.2648

****

Outcome: TB

Model Summary

R	R-sq	MSE	F	df1	df2	p
.6543	.4281	.2889	162.0719	2.0000	433.0000	.0000

Model

	coeff	se	t	p	LLCI	ULCI
constant	4.2526	.2604	16.3298	.0000	3.7408	4.7645
WFC	.3930	.0412	9.5471	.0000	.3121	.4739
KOC	-.6518	.0550	-11.8588	.0000	-.7598	-.5438

************************** TOTAL EFFECT MODEL ****************************

Outcome: TB

Model Summary

R	R-sq	MSE	F	df1	df2	p
.5547	.3077	.3489	192.9229	1.0000	434.0000	.0000

Model

	coeff	se	t	p	LLCI	ULCI
constant	5.8446	.2198	26.5875	.0000	5.4126	6.2767
KOC	-.8033	.0578	-13.8897	.0000	-.9169	-.6896

***************** TOTAL, DIRECT, AND INDIRECT EFFECTS ********************

Total effect of X on Y

Effect	SE	t	p	LLCI	ULCI
-.8033	.0578	-13.8897	.0000	-.9169	-.6896

Direct effect of X on Y

Effect	SE	t	p	LLCI	ULCI
-.6518	.0550	-11.8588	.0000	-.7598	-.5438

Indirect effect of X on Y

	Effect	Boot SE	BootLLCI	BootULCI
WFC	-.1515	.0297	-.2157	-.0980

******************** ANALYSIS NOTES AND WARNINGS *************************

Number of bootstrap samples for bias corrected bootstrap confidence intervals:
1000

Level of confidence for all confidence intervals in output:
95.00

NOTE: Some cases were deleted due to missing data. The number of such cases was:
1

------ END MATRIX -----

Mediating effect of WIF

Run MATRIX procedure:

***************** PROCESS Procedure for SPSS Release 2.13 ***************

Written by Andrew F. Hayes, Ph.D. www.afhayes.com
Documentation available in Hayes (2013). www.guilford.com/p/hayes3

****
Model = 4
Y = TB
X = KOC
M = WIF

Sample size
436

****
Outcome: WIF

Model Summary

R	R-sq	MSE	F	df1	df2	p
.1488	.0221	.6319	9.8263	1.0000	434.0000	.0018

Model

	coeff	se	t	p	LLCI	ULCI
constant	3.8458	.2958	13.0002	.0000	3.2644	4.4272
KOC	-.2440	.0778	-3.1347	.0018	-.3969	-.0910

****
Outcome: TB

Model Summary

R	R-sq	MSE	F	df1	df2	p
.6141	.3771	.3147	131.0535	2.0000	433.0000	.0000

Model

	coeff	se	t	p	LLCI	ULCI
constant	4.9401	.2461	20.0754	.0000	4.4565	5.4238
WIF	.2352	.0339	6.9427	.0000	.1686	.3018
KOC	-.7459	.0555	-13.4296	.0000	-.8551	-.6367

************************** TOTAL EFFECT MODEL ****************************
Outcome: TB

Model Summary

R	R-sq	MSE	F	df1	df2	p
.5547	.3077	.3489	192.9229	1.0000	434.0000	.0000

Model

	coeff	se	t	p	LLCI	ULCI
constant	5.8446	.2198	26.5875	.0000	5.4126	6.2767
KOC	-.8033	.0578	-13.8897	.0000	-.9169	-.6896

***************** TOTAL, DIRECT, AND INDIRECT EFFECTS ********************

Total effect of X on Y

Effect	SE	t	p	LLCI	ULCI
-.8033	.0578	-13.8897	.0000	-.9169	-.6896

Direct effect of X on Y

Effect	SE	t	p	LLCI	ULCI
-.7459	.0555	-13.4296	.0000	-.8551	-.6367

Indirect effect of X on Y

	Effect	Boot SE	BootLLCI	BootULCI
WIF	-.0574	.0222	-.1033	-.0163

******************** ANALYSIS NOTES AND WARNINGS *************************

Number of bootstrap samples for bias corrected bootstrap confidence intervals:
1000

Level of confidence for all confidence intervals in output:
95.00

NOTE: Some cases were deleted due to missing data. The number of such cases was:
1

------ END MATRIX -----

Mediating effect of FIW

Run MATRIX procedure:

***************** PROCESS Procedure for SPSS Release 2.13 ***************

Written by Andrew F. Hayes, Ph.D. www.afhayes.com
Documentation available in Hayes (2013). www.guilford.com/p/hayes3

****
Model = 4
Y = TB
X = KOC
M = FIW

Sample size
436

****
Outcome: FIW

Model Summary

R	R-sq	MSE	F	df1	df2	p
.3553	.1262	.4620	62.6949	1.0000	434.0000	.0000

Model

	coeff	se	t	p	LLCI	ULCI
constant	4.2569	.2530	16.8285	.0000	3.7597	4.7541
KOC	-.5269	.0665	-7.9180	.0000	-.6577	-.3961

****
Outcome: TB

Model Summary

R	R-sq	MSE	F	df1	df2	p
.6465	.4179	.2940	155.4599	2.0000	433.0000	.0000

Model

	coeff	se	t	p	LLCI	ULCI
constant	4.3685	.2594	16.8398	.0000	3.8587	4.8784
FIW	.3468	.0383	9.0550	.0000	.2715	.4220
KOC	-.6206	.0568	-10.9261	.0000	-.7322	-.5089

************************** TOTAL EFFECT MODEL ****************************
Outcome: TB

Model Summary

R	R-sq	MSE	F	df1	df2	p
.5547	.3077	.3489	192.9229	1.0000	434.0000	.0000

Model

	coeff	se	t	p	LLCI	ULCI
constant	5.8446	.2198	26.5875	.0000	5.4126	6.2767
KOC	-.8033	.0578	-13.8897	.0000	-.9169	-.6896

***************** TOTAL, DIRECT, AND INDIRECT EFFECTS ********************

Total effect of X on Y

Effect	SE	t	p	LLCI	ULCI
-.8033	.0578	-13.8897	.0000	-.9169	-.6896

Direct effect of X on Y

Effect	SE	t	p	LLCI	ULCI
-.6206	.0568	-10.9261	.0000	-.7322	-.5089

Indirect effect of X on Y

	Effect	Boot SE	BootLLCI	BootULCI
FIW	-.1827	.0310	-.2483	-.1282

******************** ANALYSIS NOTES AND WARNINGS *************************

Number of bootstrap samples for bias corrected bootstrap confidence intervals:
1000

Level of confidence for all confidence intervals in output:
95.00

NOTE: Some cases were deleted due to missing data. The number of such cases was:
1

------ END MATRIX -----
